# Causes, clinical features and outcomes of drug-induced liver injury in hospitalized patients in a Chinese tertiary care hospital

**DOI:** 10.1186/s40064-015-1600-8

**Published:** 2015-12-22

**Authors:** Pengcheng Ou, Yi Chen, Baozhen Li, Min Zhang, Xingyu Liu, Fangfang Li, Yi Li, Chengwei Chen, Yimin Mao, Jun Chen

**Affiliations:** Liver Diseases Center, The Second Xiangya Hospital of Central South University, Changsha, 410011 Hunan China; Normin Health Changsha Representative Office, Changsha, 410011 Hunan China; Shanghai Liver Disease Research Center of Nanjing Military Area, Shanghai, 200235 China; Division of Gastroenterology and Hepatology, Renji Hospital, School of Medicine, Shanghai Institute of Digestive Disease, Shanghai Jiao Tong University, Shanghai, 200127 China

**Keywords:** Drug-induced liver injury, Chinese herbal medicine, Incidence, Prognosis

## Abstract

Drug-induced liver injury (DILI) is an important liver disease in China, owing to the country’s huge population and the availability of a multitude of drugs. Consequently, DILI is becoming an increasingly serious health problem. However, there is not enough relevant epidemiological data, and the clinical features of these patients are not clear. We conducted this study to report the causes and clinical features of DILI in hospitalized patients, and identify the mortality and predictive factors. We retrospectively collected and analyzed the data of all hospitalized patients whose discharge diagnosis was DILI at the Second Xiangya Hospital between January 2011 and December 2014. The data analyses were performed using SAS version 9.2. Among the 469 patients who were diagnosed with DILI at discharge, 361 met the criteria for DILI on re-evaluation. The crude annual incidence rate of DILI was 92.95 cases per 100,000 patients. Chinese herbal medicine was identified as the primary cause of DILI in 36.01 % of the patients. The overall mortality was 8.59 %. Alcohol consumption, use of antituberculosis drugs, serum total bilirubin, direct bilirubin, total protein, albumin, thrombinogen time, international normalized ratio, and the model for end-stage liver disease (MELD) score were significantly correlated with DILI-associated mortality. Among them, the MELD score and albumin were found to be independent predictors of outcome in patients with DILI. Chinese herbal medicine was the primary cause of DILI in the identified patients. The MELD score and albumin were independent predictors of outcome in patients with DILI.

## Background

Drug-induced liver injury (DILI) is a serious condition that is characterized by varying degrees of liver injury. A variety of drugs can lead to various types of acute and chronic liver injury, and even severe or acute liver failure. In recent years, DILI has become one of the major liver diseases in many countries, even in the West; it is the leading cause of acute liver failure, with a survival rate of only 20 % in the absence of liver transplantation (Ostapowicz et al. 2002; Lee 2003). Statistical data from Iceland has shown that the crude annual incidence rate of DILI was 19.1 cases per 100,000 inhabitants: 75 % of the DILI cases were caused by a single prescription medication, 16 % by dietary supplements, and 9 % by multiple agents (Björnsson et al. 2013). In Asia, herbal remedies are widely used and accepted as safe and effective medication, but they are often associated with high incidences of DILI. Data from Korea and Singapore show that herbs are the primary cause of DILI (Suk et al. 2012; Wai 2006). In China, because of the huge population and multitude of drugs available, especially, the vast number of Chinese herbal medicines, DILI is becoming an increasingly serious health problem. Although, a systematic analysis of the Chinese literature showed that tuberculostatics and complementary and alternative medicines are the most common etiologies of DILI in China (Zhou et al. 2013), but the relevant clinical features and outcomes of DILI are not mentioned. To further clarify the causes, clinical features, and outcomes of DILI in hospitalized patients; we conducted this study by retrospectively collecting the four-year data of hospitalized patients diagnosed with DILI.

## Patients and methods

### Patients

We performed a retrospective study and collected data from all the hospitalized patients whose diagnosis at discharge was DILI at the Second Xiangya Hospital (Hunan, China), who were hospitalized between January 1, 2011 and December 31, 2014. Informed consent was waived due to the retrospective nature of the study. Patients were included if their diagnosis at discharge was “DILI,” “drug-induced hepatitis,” “drug-induced liver disease,” “drug-induced liver failure,” or other diagnostic terminology that suggested some form of DILI. Patients were excluded (1) if their clinical information was incomplete, and therefore the diagnosis of DILI could not be confirmed, and (2) if DILI was ruled out after a re-evaluation.

The following data were collected: (1) general information (gender, age, occupation, nationality, height, weight, etc.); (2) diagnosis at admission and discharge, disease history (including history of allergies), and drinking history; (3) information about the drug suspected to have caused the liver injury; (4) symptoms and signs; (5) results of biochemical examinations, including alanine aminotransferase (ALT), aspartate aminotransferase (AST), serum total bilirubin (TBIL), direct bilirubin (DBIL), albumin (ALB), globulin (GLO), thrombinogen time (PT), international normalized ratio (INR), alkaline phosphatase (ALP), and creatinine (Cr), and routine blood examination results the first time DILI was diagnosed and at later examinations; (7) results of laboratory tests for other liver diseases (including HAV, HBV, HCV, HDV, HEV, EBV, CMV, and herpes virus infection, Wilson’s disease, autoimmune hepatitis, etc.); (8) imaging and endoscopic results; (9) results of liver histological examination; (10) severity and prognosis of DILI.

### Diagnosis and evaluation of DILI

After the data were collected, we re-diagnosed all the patients according to the ACG clinical guidelines for the diagnosis and management of idiosyncratic DILI (Chalasani et al. 2014). Briefly, the R value was calculated (R value = Serum [ALT/ALT upper limits of normal (ULN)] ÷ [ALP/ALP ULN]), and Roussel Uclaf Causality Assessment Method (RUCAM) was performed. Patients with RUCAM scores less than six were excluded, and those with RUCAM scores greater than or equal to six were retained for further analysis. The type of DILI was determined according to the R value: it was hepatocellular if the R value was >5.0, cholestatic if the R value was <2.0, and mixed if the R value was in the range of 2.0–5.0 (Chalasani et al. 2014). Acute liver failure is defined as evidence of coagulation abnormality, usually an INR >1.5, and any degree of mental alteration (encephalopathy) in a patient without pre-existing cirrhosis and with an illness of less than 26 weeks’ duration (Polson 2005). The MELD score was calculated using the following formula (Said et al. 2004): 3.78 (Ln serum bilirubin [mg/dl]) + 11.2 (Ln INR) + 9.57 (Ln serum creatinine [mg/dl]) + 6.43. Hy’s law cases have been defined as drug-induced liver injury resulting in increased alanine aminotransferase (ALT) levels greater than three times ULN and TBIL levels greater than two times ULN after excluding other potential causes. To exclude cholestatic or mixed cases, the guidance for clinical trials states that for a Hy’s law case the liver injury should not have a significant ALP increase reflecting a cholestatic component (Temple 2006). Cases in which the ALT and TBIL corresponded to these values were classified as Hy’s cases.

### Statistical analyses

Categorical variables were expressed as frequencies and percentages. Continuous variables were presented as median (range). Variables were analyzed using univariate and multivariate logistic regression analysis for dichotomous outcomes. Odds ratios (ORs) and 95 % confidence intervals (CIs) were calculated from the confidence and standard errors of the model. Statistical significance was defined as a two-sided *p* value less than 0.05. The data analyses were performed using SAS version 9.2 (SAS Institute Inc., Cary, NC, USA).

## Results

### Incidence rate of DILI

The total number of 4-year hospitalized patients was 388,365, and 469 (120.76/100,000) of them were diagnosed with DILI. Based on the RUCAM causality assessment, 361 patients met the criteria for DILI, so the remaining 108 patients were excluded. The crude annual incidence rate of DILI in the hospitalized patients was 92.95 cases per 100,000 patients. According to the age distribution of all the DILI patients (range, 1–90 years), the DILI incidence in age group 0–10, 11–20, 21–30, 31–40, 41–50, 51–60,61–70,71–80 and 81–90 was 1.11, 2.49, 12.37, 14.96, 25.49, 20.22,14.13,7.75 and 1.39 %, respectively. The age group with the highest incidence rate (25.49 %) was found to be 41–50 years and the second highest incidence rate (20.22 %) was found to be 51–60 years.

### Baseline characteristics

Table 1 represents the demographic data, clinical features and laboratory findings of the patients the first time they were diagnosed with DILI. Of the 316 patients, 195 were male and 110 were female, and their median age was 49 years (range, 37–59). Most of the patients had hepatocellular DILI (63.16 %), while 13.85 % had cholestatic DILI and 13.30 % had mixed-type DILI. The remaining patients (9.70 %) were not examined for ALP when they were diagnosed with DILI the first time, so the R value could not be calculated. Further, 32 (8.86 %) patients were HBsAg positive and 24 (6.65 %) were positive for the autoimmune hepatitis antibody; all the patients were negative for HCV, HAV, HEV, HDV and other hepatotropic viruses. Alcoholic liver disease was diagnosed in 22 (6.09 %) patients, and NAFLD was diagnosed in 13 (3.60 %) patients. Wilson’s diseases and other liver diseases were not diagnosed in any of the patients. The total number of patients with pre-existing liver disease was 91 (25.21 %), pre-existing decompensated liver diseases were not found in these patients.

**Table 1:** Baseline characteristics of the 361 patients

Characteristics	Total (N = 361)
Age (y)	49 (37, 59)^a^
Gender (M/F), n (%)	195 (54 %)/166 (46 %)^b^
Alcohol consumption, n (%)	33 (9.14 %)^b^
Pre-existing liver disease, n (%)	91 (25.21 %)^b^
HBV, n (%)	32 (8.86 %)^b^
Autoimmune antibodies, n (%)	24 (6.65 %)^b^
Alcoholic liver disease, n (%)	22 (6.09 %)^b^
NAFLD, n (%)	13 (3.60 %)^b^
Days from drug use to symptom appearance	30 (9, 60)^a^
Days from drug discontinuation to symptom	
Disappearance	20 (12, 30)^a^
ALT (U/L)	225.7 (89.7, 541.3)^a^
AST (U/L)	125.8 (57.1, 325.1)^a^
ALP (U/L)	135.1 (81.7, 203.9)^a^
TBIL (μmol/L)	53 (14.5, 237.1)^a^
DBIL (μmol/L)	38.1 (6.7, 182.9)^a^
PT (s)	13.3 (12, 16.6)^a^
INR	1 (1, 1.4)^a^
TP (g/L)	53 (14.5, 237.1)^a^
ALB (g/L)	33.4 (29.5, 36.8)^a^
Cr (μmol/L)	56.1 (45.8, 69)^a^
MELD	6.9 (0.1, 13.3)^a^
Type of DILI	
Hepatocellular, n (%)	228 (63.16 %)^b^
Cholestatic, n (%)	50 (13.85 %)^b^
Mixed, n (%)	48 (13.30 %)^b^
NA, n (%)	35 (9.70 %)^b^

*HBV* Hepatitis B virus, *NAFLD* non-alcoholic fatty liver disease, *ALT* alanine transaminase, *AST* aspartate transaminase, *ALP* alkaline phosphatase, *TBIL* total bilirubin, *DBIL* bilirubin direct, *PT* prothrombin time, *INR* international normalized ratio, *TP* total protein, *ALB* albumin, *Cr* Creatinine, *MELD* model for end-stage liver disease

^a^ OR (95 % CI)

^b^ n (%)

### Main clinical manifestations

The digestive symptoms, including nausea, anorexia, vomiting and abdominal distension, were observed in most patients (59.28 %). Jaundice was also one of the main clinical symptoms (52.63 %). Itching occurred in 13.30 % of the patients, fever in 15.51 %, and rash in 8.31 %. Severe liver damage symptoms including hemorrhage tendency and hepatic encephalopathy occurred in 3.05 % of the patients. In 21.61 % of the patients, the results of liver biochemical examination were abnormal, but there were no symptoms.

### Causative drugs

The number of DILI cases associated with each type of drug is shown in Table 2. We found Chinese herbal drugs to be the leading cause of DILI (36.01 %). Further, antithyroid and antituberculosis drugs accounted for more than 14 % of the DILI cases. As shown in Table 3, the most commonly implicated Chinese herbal medicine were radix polygoni multiflori, panax pseudo-ginseng, *Tripterygium wilfordii*, saffron, shenbao mixture, and decoction of herbal medicine ingredients; the major implicated antithyroid were propylthiouracil and methimazole; rifampicin, isoniazid and pyrazinamide were the major implicated antituberculosis. Antituberculosis and chemotherapy drugs were often involved in a combination at least two same class drugs.

**Table 2:** Causative drugs

Drug	Cases (n)	Percentage (%)
Chinese herbal medicine	130	36.01
Antithyroid	52	14.4
Antituberculosis	51	14.13
Antibiotics	26	7.2
Chemotherapy drugs	22	6.09
Immunosuppressants	22	6.09
Antipyretics and analgesics	13	3.6
Psychotropic drugs	11	3.05
Antidiabetics	9	2.49
Lipid-lowering drugs	8	2.22
Others	17	4.71

Others include heroin, edaravone, omeprazole, thyroxine and amlodipine besylate, each of which was associated with less than four cases of DILI

**Table 3:** Specific drug of causing DILI

Category	Specific drug	Cases (n)	Percentage (%)
Chinese herbal medicine	Herbal medicine ingredients	91	70.00
	Radix polygoni multiflori	15	11.54
	*Panax* pseudo-ginseng	10	7.69
	*Tripterygium wilfordii*	6	4.62
	Saffron	4	3.08
	And shenbao mixture	4	3.08
Antithyroid	Propylthiouracil	26	50
	Methimazole	26	50
Antituberculosis	Isoniazid + rifampicin + pyrazinamide	29	56.86
	Isoniazid + rifampicin	17	33.33
	Rifampicin	4	7.85
	Isoniazid + pyrazinamide	1	1.96
Antibiotics	Quinolones	7	26.92
	Cephalosporin	6	23.08
	United	4	15.38
	Acyclovir	3	11.53
	Vancomycin	2	7.70
	Meropenem	2	7.70
	Biaxin	2	7.70
Chemotherapy drugs	Docetaxel + platinum	10	45.45
	Platinum	4	18.18
	Etoposide	4	18.18
	Gemcitabine	3	13.64
	Imatinib	1	4.55
Immunosuppressants	Leflunomide	6	27.27
	Methotrexate	5	22.73
	Acipimox	3	13.64
	Thalidomide	3	13.64
	Cyclosporin	3	13.64
	Phenylbutazone	2	9.09
Antipyretics and analgesics	Acetyl aminophenol	9	69.23
	Meloxicam	2	15.38
	Celecoxib	2	15.38
Psychotropic drugs	Clozapine	3	27.27
	Olanzapine	2	18.18
	Carbamazepine	2	18.18
	Dilantin sodium	2	18.18
	Lithium carbonate	2	18.18
Antidiabetics	Metformin	5	55.56
	Gliclazide	2	22.22
	Acarbose	2	22.22
Lipid-lowering drugs	Statins	8	100

### Severity and prognosis of DILI

Among the 361 patients, 91.41 % survived. Among the 31 (8.59 %) patients who died, 22 (70.97 %) died as a result of severe liver disease, 9 (29.03 %) as a result of primary diseases, and 10 (32.26 %) died from anti-TB drugs hepatoxity. According to Hy’s criteria, 71 (19.67 %) patients were classified under Hy’s patients: 9 (12.68 %) of these patients died from liver failure (Table 4).

**Table 4:** Severity and prognosis of DILI

Prognosis	Cases (n)	Percentage (%)
Survival	330	91.41
Death	31	8.59
Cause of death		
Liver diseases	22	70.97
Other diseases	9	29.03
Died from anti-TB		
Drugs hepatoxity	10	32.26
Hy’s cases	71	19.67
Mortality in Hy’s cases	9	2.68

### Logistic regression analysis

**Fig. 1:**
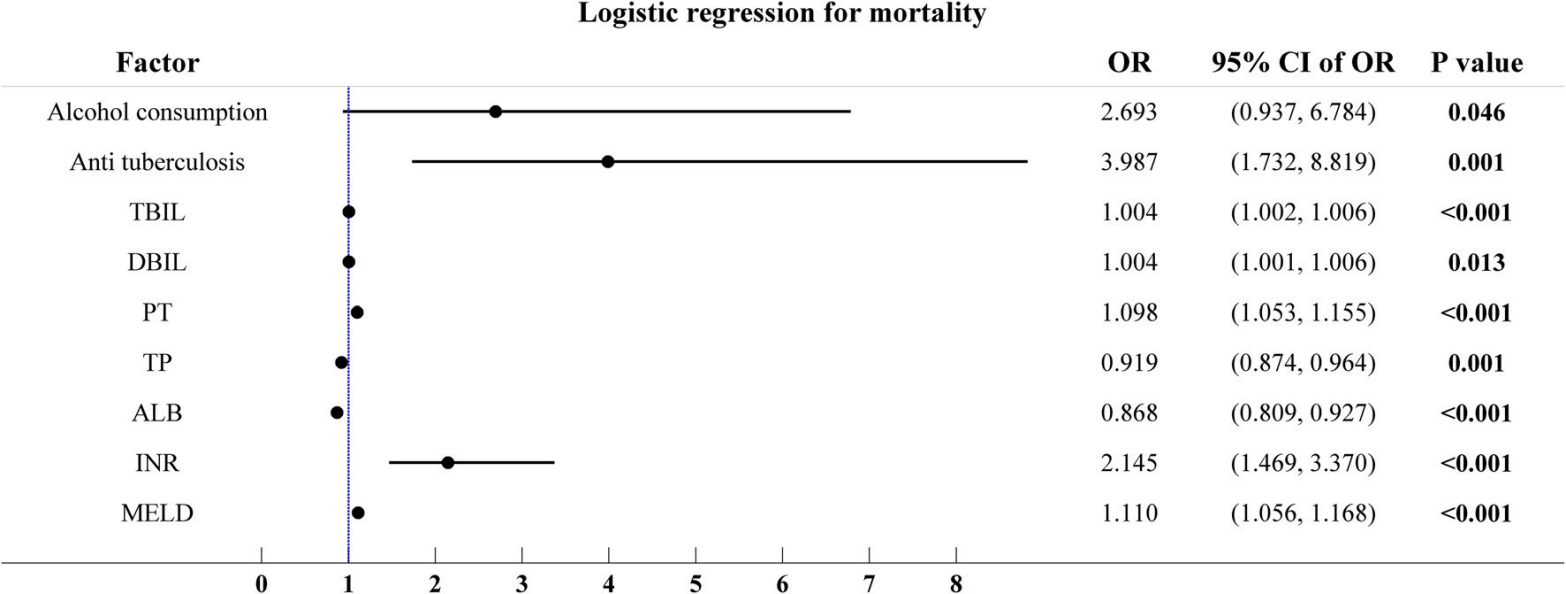
Logistic regression analysis. *TBIL* total bilirubin, *DBIL* direct bilirubin, *TP* total protein, *ALB* albumin, *PT* thrombinogen time, *INR* international normalized ratio, *MELD* model for end-stage liver disease, *OR* odds ratios, *CI* confidence intervals

Univariate logistic regression analysis showed that alcohol consumption, use of antituberculosis drugs, serum TBIL, DBIL, total protein, ALB, PT, INR and the MELD score were significantly associated mortality (*p* < 0.05) (Table 5). The correlation coefficient values indicated that alcohol consumption, the use of antituberculosis drugs, serum TBIL, DBIL, PT, INR and the MELD score were positively correlated with mortality, while the total protein and ALB were negatively correlated with mortality. The hazard ratio showed that among all the predictors, use of antituberculosis drugs was the most hazardous factor correlated with the mortality rate, followed by alcohol consumption and then INR (Fig. 1). Multivariate logistic regression analysis showed that MELD and ALB were independent predictors of poor outcomes (*p* < 0.05).

**Table 5:** Logistic regression analysis

Predictor	Univariate analysis		Multivariate analysis	
	95 % CI	*p* value	95 % CI	*p* value
Male	0.495–2.203	0.923		
Female	0.454–2.019	0.923		
Age (y)	0.996–1.045	0.110		
Alcohol consumption	0.937–6.784	***0.046***	0.718–11.505	0.136
Pre-existing liver disease	0.935–4.46	0.073		
Causative drugs				
Chinese herbal medicine	0.438–2.070	0.949		
Antithyroid	0.249–2.350	0.804		
Antituberculosis	1.732–8.819	***0.001***	0.270–82.810	0.288
Antibiotics	0.137–3.173	0.879		
Chemotherapy drugs	0.166–3.918	0.986		
Antipyretics and analgesics	0.048–4.723	0.883		
Days from drug use to symptom appearance	0.999–1.000	0.992		
Days from drug discontinuation to symptom disappearance	0.984–1.024	0.268		
ALT (U/L)	1.000–1.000	0.425		
AST (U/L)	1.000–1.001	0.281		
ALP (U/L)	0.997–1.001	0.838		
TBIL (μmol/L)	1.002–1.006	**<** ***0.001***	0.990–1.012	0.874
DBIL (μmol/L)	1.001–1.006	***0.013***	0.982–1.011	0.602
PT	1.053–1.155	**<** ***0.001***	0.875–1.071	0.531
INR	1.469–3.370	**<** ***0.001***	0.713–2.600	0.350
TP (g/L)	0.874–0.964	***0.001***	0.915–1.066	0.744
ALB (g/L)	0.809–0.927	**<** ***0.001***	0.772–0.983	***0.025***
Cr (μmol/L)	0.997–1.004	0.369		
MELD	1.056–1.168	**<** ***0.001***	1.004–1.192	*** 0.041***
Type of DILI				
Hepatocellular	0.375–1.671	0.539		
Cholestatic	0.791–4.792	0.147		
Mixed	0.322–2.885	0.527		
Hy’s case	0.197–1.719	0.327		

The bold italic data reflected significant difference

*ALT* alanine transaminase, *AST* aspartate transaminase, *ALP* alkaline phosphatase, *TBIL* total bilirubin, *DBIL* bilirubin direct, *PT* thrombinogen time, *INR* international normalized ratio, *TP* total protein, *ALB* albumin, *Cr* Creatinine, *MELD* model for end-stage liver disease

## Discussion

Our statistical data indicated that the crude annual DILI incidence in hospitalized patients was 92.95/100,000. This is higher than the incidence rate reported in Iceland (19.1/100,000 inhabitants) (Björnsson et al. 2013). Certainly, the study population is different and may be the reason for the difference, especially as the drug use in hospitalized patients may be higher. As there is a lack of epidemiological data for it, the DILI incidence in the general population of China is not known.

In hospitalized patients, misdiagnosis and missed diagnosis are common, and there is still no standard diagnostic criteria for DILI in China. Most of the diagnoses are based on the physicians’ individual ability and experience, and the RUCAM causality assessment is seldom used. Sometimes, because of conflicts with the hospital administration, physicians often refuse to diagnose DILI, which leads to a missed diagnosis. Therefore, there is a clear need to establish standard diagnostic criteria and reporting guidelines for DILI.

It is generally believed that the incidence of DILI is higher in patients aged over 50 years and that the incidence of DILI increases with age (Björnsson et al. 2013; Chalasani et al. 2014). However, in our group of hospitalized patients, the incidence was the highest in the 41- to 50-year age group. However, it should be noted that this does not reflect its incidence in the general population.

In China, Chinese herbal medicine is broadly accepted as safe and effective medication for the treatment of various ailments. The diagnosis and treatment strategies of traditional Chinese medicine are completely different from those of Western medicine. In traditional Chinese medicine, the practice is experienced-based rather than evidence-based. Therefore, very often, there is not enough evidence to prove the treatment and side effects of a drug. Our data suggest that the use of Chinese herbal medicine was associated with more than one-third of the DILI cases (36.01 %) in all the hospitalized patients diagnosed with DILI. In Korea and Singapore too, herbal medicine has been reported to be the leading cause of DILI (Suk et al. 2012; Wai 2006). Another group of data from Shanghai (China) also confirmed that Chinese herbal medicine accounted for 53.62 % of DILI cases in hospitalized patients diagnosed with DILI (Lai et al. 2012). Thus, it is important to monitor the hepatotoxicity of Chinese herbal medicine. This is especially important as the dosage of herbal drugs and their compositions vary between these traditional practitioners, and it is therefore very difficult to determine which component or, rather, components is the cause of the liver injury. Currently, only a few components of Chinese herbal medicine are known to cause DILI. There seems to be an urgent need to standardize the compositions of herbal medication and provide guidelines for the dosage.

The incidence of hyperthyroidism and tuberculosis is fairly high in China, which is reflected in our results: antithyroid and antituberculosis drugs were found to be the second and third most hepatotoxic drugs. Recently, a nationwide retrospective epidemiological investigation on DILI has been undertaken by a study group, with more than 300 hospitals participating; the results of this study will be highly useful for clarifying the DILI situation in the general population of China.

According to our data, the prognosis of most DILI patients was good, and the survival rate was 91.41 %. The manifestation was mild in most of the patients, and some patients were even asymptomatic. Further, the liver function of the patients rapidly improved after the hepatotoxic drugs were discontinued. These findings suggest that early detection of abnormal liver function and timely discontinuation of the drugs are very necessary. Generally, the mortality rate of DILI has been reported to be 8–17 % (Larrey and Pageaux 2005; Andrade et al. 2005; Chalasani et al. 2008; Devarbhavi et al. 2010). In our study, the mortality rate was 8.59 %. According to Hy’s law, if hepatocellular injury causes jaundice in a patient during a phase three trial, have at least a 10 % mortality rate (Temple 2006). In our investigation, the mortality rate in Hy’s cases was 12.69 %, these findings confirmed Hy’s theory is an important indicator of the potential of a drug to cause serious liver injury. This observation was also confirmed in some large studies on DILI in Spain (Andrade et al. 2005) and in Sweden (Björnsson et al. 2005) in which 10 % of the subjects with hyperbilirubinemia or jaundice died or needed liver transplants.

The risk factors for mortality were baseline alcohol consumption, the use of antituberculosis drugs, TBIL, DBIL, PT, INR MELD score, TP and ALB. Among these risk factors, alcohol consumption was associated with poor outcome. However, there is not enough evidence to indicate that chronic alcohol consumption is a risk factor for all-cause DILI. It has been reported that heavy alcohol consumption is a risk factor for DILI owing to the presence of compounds such as APAP, methotrexate, and isoniazid (Chalasani et al. 2014).

The other risk factor for mortality is antituberculosis drugs, as it is well known that antituberculosis drugs such as rifampicin, isoniazid and pyrazinamide have strong hepatotoxicity. A study from India revealed acute liver failure occurred in a quarter of DILI patients receipted antituberculosis treatment and the overall motality was 22.7 % (Devarbhavi et al. 2013). Our data also indicate that antituberculosis drugs caused 32.26 % of the mortalities, which suggested that the hepatoxicity of antituberculosis medication is a serious problem among patients undergoing antituberculosis treatment. A survey from Taiwan followed 926 patients for 4122.9 person-months (pm) and found that 111 (12.0 %) developed hepatotoxicity after a median of 38.0 days from the start of treatment; moreover, the severe hepatotoxicity rate was 3.5 %. The independent risk factors for hepatotoxicity were old age, female sex, autoimmune disease, human immunodeficiency virus infection, higher pyrazinamide dosage in the last 8–14 days, and lower rifampicin dosage in the last 15–21 days (Shu et al. 2013).

The other risk factors for mortality were TBIL, DBIL, TP, ALB, PT, INR and the MELD score, which reflect the state of the liver parenchyma. Compared to ALT, AST or ALP, these risk factors can more objectively and accurately reflect the degree of damage to the liver parenchyma. Increase in TBIL, DBIL, PT, INR and the MELD score was accompanied by an increase in mortality; this is expected, as increase in these biochemical indices is one of the criteria for diagnosis of liver dysfunction. On the other hand, increase in the level of some indicators such as ALB was accompanied with a decrease in mortality. Our results also show that sex, age, latencies and DILI type are not correlated with DILI-associated mortality. This finding is consistent with studies from the USA (Chalasani et al. 2015) and Korea (Jeong et al. 2015). The data from the US study showed that the DILI-associated mortality was significantly higher in individuals with pre-existing liver disease (Chalasani et al. 2015). Although our results showed that pre-existing disease is not a risk factor for DILI-associated mortality (p = 0.073), the results did indicate that pre-existing liver disease could enhance the mortality. This needs to be examined in a larger population in order to confirm the results.

Our study found that the MELD score and ALB were independently associated with poor outcome. Thus, the MELD score and ALB are key risk factors for DILI-associated mortality. The MELD score is widely accepted as an accurate predictor of mortality across a broad spectrum of liver diseases (Said et al. 2004; Martin et al. 2014). The study from Korea also confirmed that the c-statistic for the MELD score alone was 0.93 and that the MELD score had strong discriminatory power and may be a reliable predictor of the prognosis in patients with DILI (Jeong et al. 2015).

One of the main limitations of our study is its nature, this study being a single-center retrospective study. Another limitation was that some important examinations such as liver biopsy were not performed in most of the patients, and the data for some important biochemical indexes were missing from the first time the patients were diagnosed with DILI. Moreover, we did not consider the effects of drug interaction in cases where multiple drugs were used, as these interactions are quite complicated and unclear. In China, several drugs for liver protection are used in cases of DILI, usually at least two different liver protection drugs for one patient. This makes it difficult to compare the therapeutic effects of these drugs, so we did not analyze the treatment data in our study.

In summary, Chinese herbal medicine was the main cause of DILI in hospitalized patients in China, followed by antithyroid and antituberculosis drugs. The survival rate was 91.41 %. The major cause of mortality was severe liver diseases, and primary diseases were the other cause. DILI associated with the use of antituberculosis drugs was the cause in 32.26 % of the patients. Further, alcohol consumption, the use of antituberculosis drugs, serum TBIL, DBIL, TP, ALB, PT, INR and the MELD score were significantly correlated with DILI-associated mortality. Among them, the MELD score and ALB were independent predictors of outcome in patients with DILI.

## Ethics approval

The local ethics committee, which acts as the institutional review board of the Second Xiangya Hospital of Central South University.
